# Descending aortic transection for recurrence of a pseudoaneurysm previously treated with a stent graft after extra-anatomical bypass for aortic coarctation: a case report

**DOI:** 10.1186/s40792-021-01136-4

**Published:** 2021-02-16

**Authors:** Keisuke Shibagaki, Shingo Kunioka, Yuta Kikuchi, Naohiro Wakabayashi, Tomonori Shirasaka, Natsuya Ishikawa, Hiroyuki Kamiya

**Affiliations:** Department of Cardiac Surgery, Asahikawa Medical University, Midorigaoka Higashi 2-1-1-1, Asahikawa, 078-8510 Japan

**Keywords:** Coarctation of the aorta, Extra-anatomical bypass grafting, Pseudoaneurysm, Aortic transection, Open repair

## Abstract

**Background:**

In adult patients with primary or recurrent coarctation of the aorta (CoA), extra-anatomic bypass grafting (EABG) has been widely used as a surgical treatment option. However, there have been few reports on pseudoaneurysms (PAs) of the distal anastomotic sites after extra-anatomic bypass for CoA.

**Case presentation:**

A 51-year-old man with hemoptysis was transferred to our hospital. Twenty years ago, he had undergone EABG from the ascending to the descending aorta (ascending-to-descending EABG) for CoA with right aortic arch. Eight years ago, he underwent thoracic endovascular aortic repair (TEVAR) for the ruptured PA on the distal anastomotic site of the EABG. Contrast-enhanced computed tomography scans revealed recurrent ruptured PA on the distal anastomotic site of the EABG. Therefore, we decided to replace the descending aorta, followed by end-to-side anastomosis of the EABG to the replaced descending aorta. However, due to massive adhesion of the lung to the EABG and PA, we performed transection of the descending aorta to decompress the PA. The postoperative course was uneventful, and the patient is doing well 5 months after surgery.

**Conclusions:**

Aortic transection between the CoA and the distal anastomosis site may be a useful additional procedure in patients previously treated with TEVAR for PAs in the distal anastomosis site after EABG.

## Background

In adult patients with primary or recurrent coarctation of the aorta (CoA), extra-anatomic bypass grafting (EABG) has been widely used as a surgical treatment option [1]. However, there have been few reports on pseudoaneurysms (PAs) after EABG for CoA. Here, we report the case of a patient who was treated with proximal aortic transection for a recurrent and ruptured PA on the distal anastomotic site of EABG that had previously been treated with thoracic endovascular aortic repair (TEVAR).

## Case presentation

The patient in our case was a 51-year-old man who had been treated 20 years ago with ascending-to-descending EABG using a 22-mm Dacron graft for CoA. Moreover, he had anatomical anomalies of the right aortic arch with Kommerell’s diverticulum. Eight years ago, the patient experienced hemoptysis due to a PA on the distal anastomosis site and was treated by TEVAR using the GORE TAG Stent Graft System (34 mm × 20 cm) (W.L. Gore & Associated, Inc., Flagstaff, AZ, USA). After TEVAR, the size of the PA decreased, and the patient did not experience hemoptysis.

**Fig. 1:**
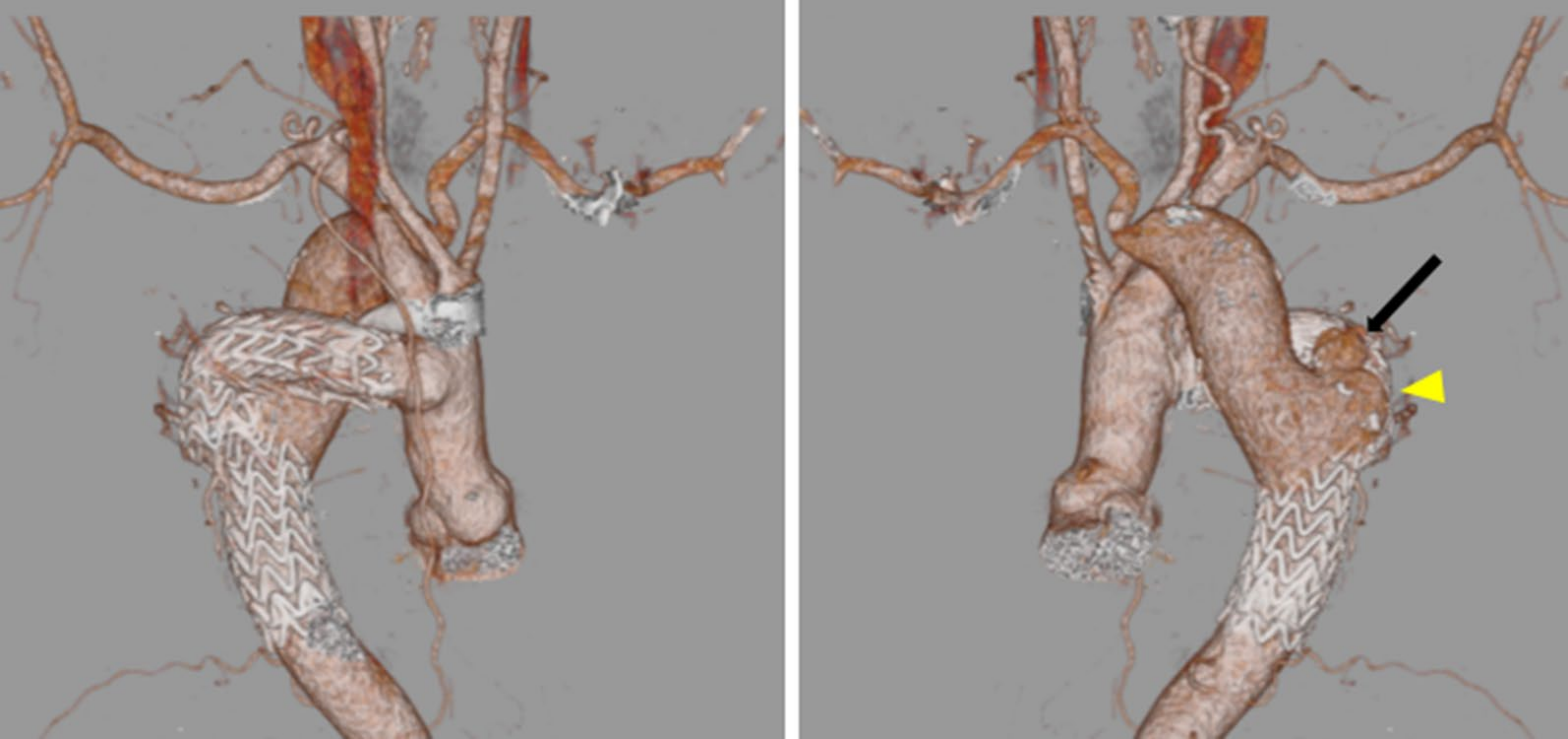
Preoperative computed tomography three-dimensional (3D) reconstruction. Anterior (**a**) and posterior (**b**) views show the right aortic arch and distal anastomotic aneurysm and contrast medium extravasation outside the aneurysm (black arrow). A small extension (similar to Kommerell diverticulum) was found in the posterior (**b**) view (yellow arrowhead)

However, due to a recurrence of hemoptysis, the patient was admitted to a hospital. On admission, contrast-enhanced computed tomography (CT) revealed the presence of a PA in the distal anastomotic site of the graft, which appeared to be the cause of hemoptysis (Fig. 1). The stent graft was observed in the distal anastomotic site, as was massive blood flow from the descending aorta. Therefore, the patient was transferred to our institution. Blood pressure was normal on examination, and we initially planned to treat the patient using TEVAR. The stent graft is ideally deployed from a site distal to the coarctation to cover the PA, but in this case, it could not be placed proximally to the PA owing to the anatomical form. Thus, we decided to replace the descending aorta and subsequently performed end-to-side anastomosis of the replaced descending aorta with the graft that was surgically constructed 20 years ago.

**Fig. 2:**
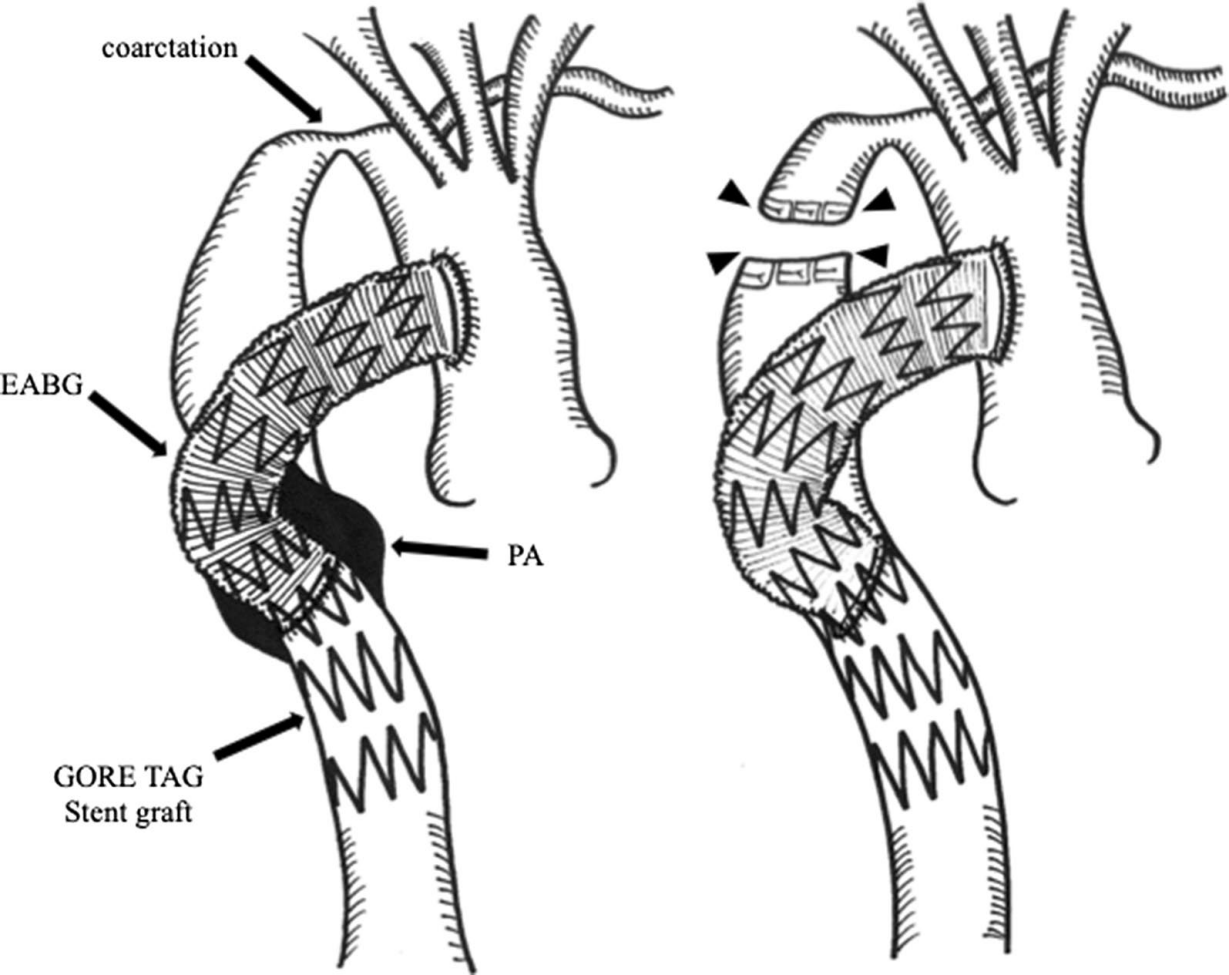
Schematic images of the surgical procedures: preoperative (**a**) and postoperative (**b**). The arrowhead shows transected site of the descending aorta

**Fig. 3:**
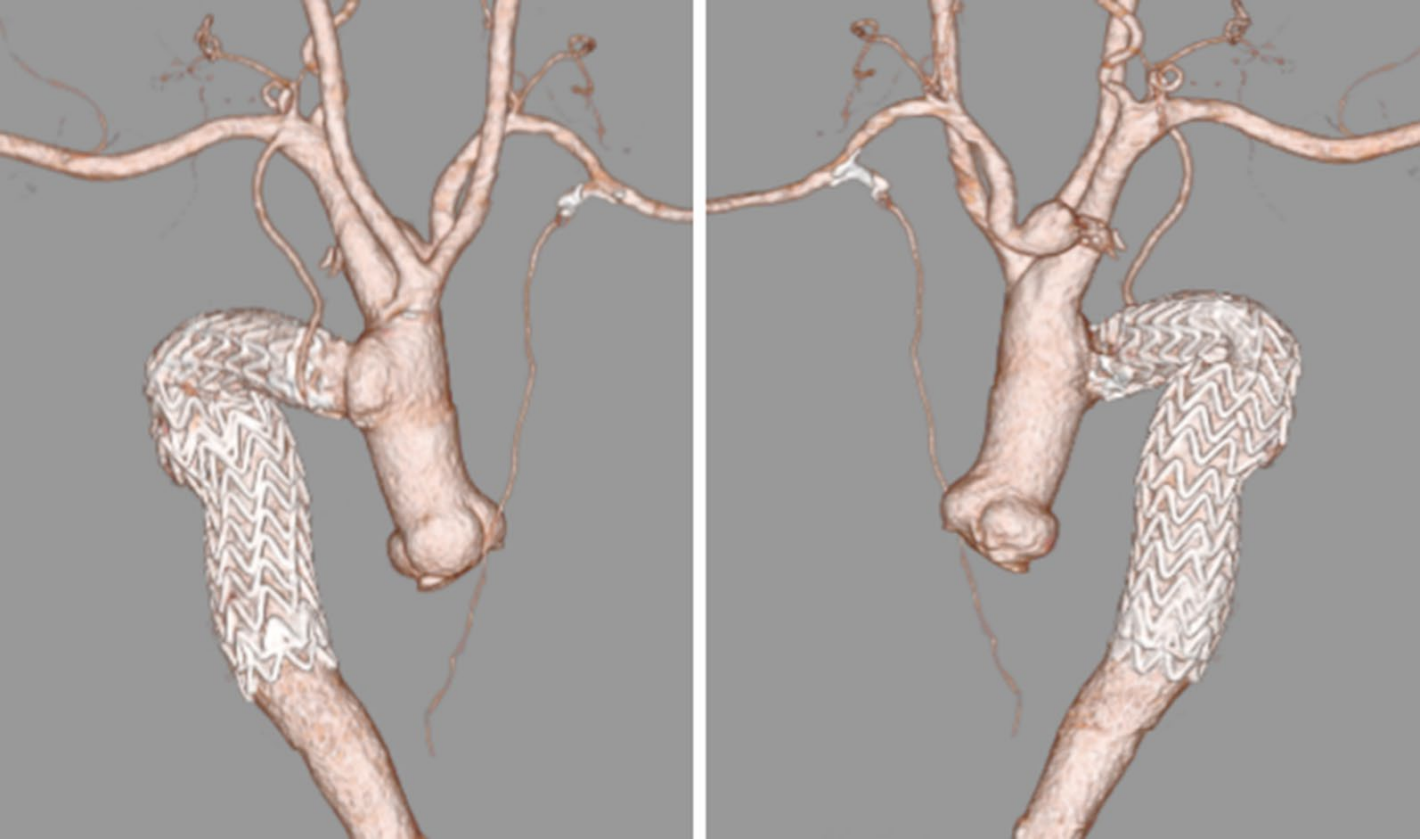
Postoperative computed tomography with 3D reconstruction. Anterior (**a**) and posterior (**b**) views show no contrast medium extravasation

The patient underwent a right third intercostal thoracotomy. Because large portions of the lung had adhered to the ascending-to-descending EABG and PA, we modified the initial surgical plan to perform replacement of the descending aorta, followed by end-to-side anastomosis of the graft to the newly replaced descending aorta. The native descending aorta proximal to the anastomosis site was accessible; therefore, we decided to completely transect the descending aorta for decompression of the PA (Fig. 2). The descending aorta was clamped between the coarctation and PA and transected. Both sides were closed using 4-0 polypropylene with pledget (ETHICON®). Postoperative contrast-enhanced CT showed the complete disappearance of the diseased aortic arch, including the ruptured PA (Fig. 3). The postoperative course was uneventful, and the patient was transferred back to the previous hospital on the 13th postoperative day to continue rehabilitation. The patient is doing very well at 5 months after the surgery.

## Discussion

This case report describes the transection of the descending aorta to treat a recurrent ruptured PA on the distal anastomotic site who was previously treated with TEVAR and had undergone ascending-to-descending EABG for CoA.

CoA is a common congenital malformation, and several surgical procedures have been used as treatment options for patients diagnosed in adolescence or adulthood [2–5]. Notably, EABG, particularly ascending-to-descending aortic bypass, has been associated with low mortality and morbidity rates [1]. Thus, EABG may be a safer and more efficacious surgical option.

Regarding complications after open surgery for CoA, Gawenda et al. reported that late formation of aneurysms could occur after every type of surgical direct repair; the CoA site wall is thin and fragile, and the incidence of late-forming aneurysms increases with time [5]. In this case, the patient had massive hemoptysis for the first time 12 years after EABG. The patient had a small Kommerell diverticulum at the orifice of the right aberrant subclavian artery. Generally, patients with Kommerell diverticulum have fragile descending aortas, and it is possible that aortic wall fragility in our patient resulted in the PA.

Nevertheless, PAs after EABG for CoA are extremely rare. Prevenzta et al. reported that true aneurysms were observed in only 0.2% of patients [6], and there have been only two cases of PAs in the anastomotic site after EABG for CoA [7, 8]. To the best of our knowledge, this is the first case report of an open repair for a PA on the distal anastomotic site after EABG for CoA.

In the present case, the PA was treated with TEVAR 12 years after the initial surgery. Anatomically, TEVAR appeared to be an incomplete operation because the blood flow from the native descending aorta was not completely stopped. However, the patient survived for 8 years after this emergency surgery, suggesting that TEVAR cannot cure PAs in the distal anastomotic site, but it can reduce the pressure on the PA. Therefore, such surgery may be justified in select patients.

This report presents a case where the descending aorta was transected to control fatal hemoptysis in an emergency setting. Surgical resection of the PA was extremely risky owing to massive adhesion, but it resulted in an unexpectedly good outcome. Our experience suggests that aortic transection may be a useful additional procedure for this rare condition.

## Conclusions

Aortic transection between the CoA and distal anastomosis site may be a useful additional procedure for PAs in the distal anastomosis site after EABG in patients previously treated with TEVAR.

## Data Availability

Data supporting the conclusions are included in the article.

## Abbreviations

CoA: Coarctation of the aorta
EABG: Extra-anatomic bypass grafting
PA: Pseudoaneurysm
TEVAR: Thoracic endovascular aortic repair
CT: Computed tomography
